# Charles Bonnet Syndrome (CBS) in a Patient With Secondary Progressive Multiple Sclerosis: A Case Report

**DOI:** 10.1002/ccr3.70588

**Published:** 2025-07-06

**Authors:** Shima Jahani, Atefeh Eidi Ardizi, MohammadAli Sahraian

**Affiliations:** ^1^ Multiple Sclerosis Research Center Neuroscience Institute, Tehran University of Medical Sciences Tehran Iran

**Keywords:** Charles bonnet syndrome, cognition, multiple sclerosis, secondary progressive multiple sclerosis

## Abstract

Here we present a 69‐year‐old woman with Secondary Progressive Multiple Sclerosis (SPMS) and Charles Bonnet Syndrome. Our case underlines how clinicians should consider this rare syndrome in cognitively intact MS patients with visual hallucinations.

## 1 Introduction

Charles Bonnet syndrome (CBS) is a very rare disease defined by occurrence of complex visual hallucinations in individuals who have experienced significant vision loss. It was first described by Charles Bonnet based on experiences of his grandfather, Monsieur Lullin who started to have visual disturbance shortly after his cataract surgery [1].

CBS patient's complex hallucinations can be diverse, including images of people, animals, or elaborate scenes. These hallucinations are reported to occur at any time of the day, specifically at nights. There is a vast variation on this conditions duration, ranging from brief incidents to episodes extending over a five‐year period. Although patients are aware that their visions are not real, the prolonged nature of their symptoms causes extreme distress and, in some cases, depression. This is reflected in their notably reduced quality of life [2].

Multiple Sclerosis (MS) is a neurologic disorder with various clinical features which could potentially cause visual problems [3, 4, 5]. Although optic neuritis has been a common companion of MS, there have been relatively few reported cases of CBS among MS patients [6, 7, 8]. Here we describe a case of CBS in an elderly patient with secondary progressive MS (SPMS).

## 2 Case Presentation

### 2.1 Case History/Examination

The patient presented here is a 69‐year‐old Iranian with SPMS, who was brought to the emergency room in an agitated state, accompanied by her personal caretaker. Since her diagnosis of MS at age 29, she experienced several attacks and progressive disability necessitating assistive devices for walking and dependency for daily tasks. From 10 years before, even though she did not experience specific attacks, her disease pattern turned progressive, converting to SPMS. Two months prior to her visit, her visual acuity worsened further, leading her to become completely bedridden. Ever since, she experienced hallucinations of spiders in various shapes and sizes walking toward her. Despite her being aware of the non‐reality nature of hallucinations, they caused her extreme anxiety and distress, resulting in her being agitated and aggressive toward her caregivers.

Our patient's caretaker did not report any recent infection or trauma. Furthermore, she reported negative history for any autoimmune diseases and neurological disorders within her family. Upon examination, she was alert and oriented to time, place, and date. Though agitated, her vital signs were within normal ranges. The systemic examination did not reveal any significant findings. Force of right and left upper limbs was four out of five, while strength in the lower limbs was significantly diminished, both one out of five. Reflexes in both upper and lower limbs were hyperactive, and a positive Babinski reflex was seen in both lower limbs.

Her pharmaceutical regimen was consisted of Xacrel (ocrelizumab) and solifenacin. Xacrels last dose was administered a week prior to her visual worsening. She did not use any illicit substances, and she had no suicidal tendencies during her hospitalization. She had no other important clinical history.

## 3 Method (Investigations, Diagnosis, and Treatment)

**FIGURE 1:**
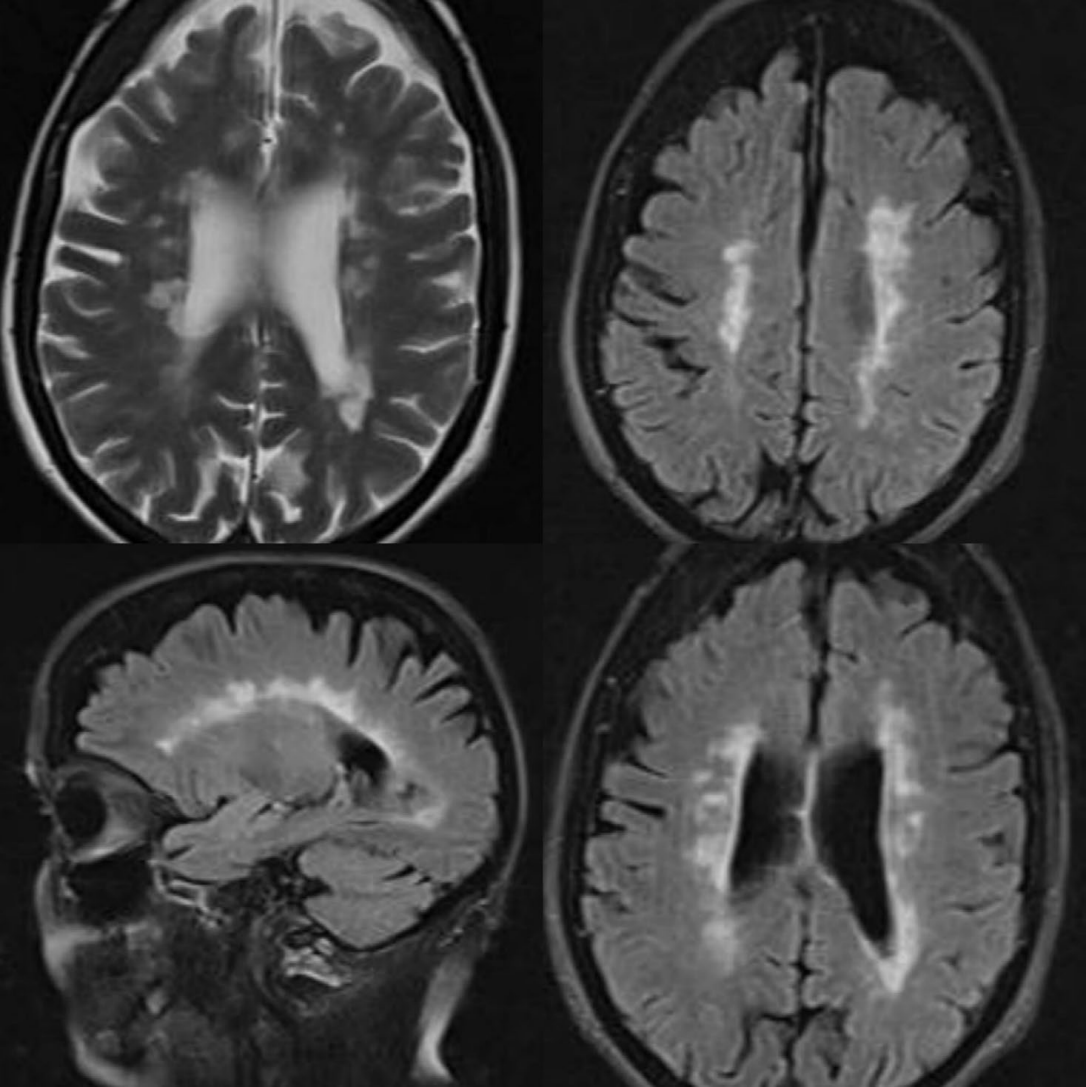
Multiple demynileiting images in periventricular region, compatible with MS.

An interdisciplinary examination by a neurologist, a psychiatrist and a physical therapist ensued. Magnetic resonance imaging (MRI) showed demyelinating plaques in the left temporal subcortical matter, left cerebellar hemisphere, and bilateral pontine regions (Figure 1). Subsequently, the multidisciplinary team of physicians reached the conclusion to diagnose the patient with CBS. The patient was treated with olanzapine 5 mg before bedtime.

## 4 Conclusion and Results (Outcome and Follow‐Up)

Seven days after her admission she was discharged with no visual hallucination and a good general condition. Subsequent follow‐up every 6 months up to 2 years revealed no symptom recurrence.

## 5 Discussion

We described a 69‐year‐old woman with a history of MS who experienced visual hallucination after worsening of her visual acuity. To our best knowledge there have been no prior report on co‐occurrence of advanced MS and CBS, even though there have been manuscripts presenting cases of CBS in the first attack of MS [9].

CBS is characterized by experiencing visual hallucinations in a visually impaired patient who is psychiatrically intact. Even though CBS is mainly reported in the context of ophthalmic conditions, recent reports support its happening in neurological conditions such as neurodegenerative diseases [10]. CBS diagnostic criteria have been a subject of debate for long, with one of the first ones being proposed by Podoll et al. with the following statements: (1) the presenting symptom should be visual hallucinations in elderly patients who are cognitively intact, (2) the hallucination should not be caused by a mental condition or neurological condition [11]. A systematic review of the CBS diagnostic criteria by Hamedani et al. [12] declared that even though hallucinations and vision loss are essential for diagnosis, the types of visual hallucination and degree of vision loss were vastly different. These findings highlight that even though a patient presenting with hallucinations should be worked up for psychological conditions, in case the patient has insight into her hallucinations and prior psychiatric conditions were absent, other neurological disorders such as CBS should be considered [12].

To explain the pathophysiology of this condition, there have been three possible explanations Perceptual Release Theory, Deafferentation and Cortical Hyperexcitability and sensory deprivation theory. In perceptual release theory its suggested that visual hallucinations in CBS patients occur when sensory input from the eyes is reduced, causing the brain to “release” stored images or memories that are normally suppressed. The second theory named deafferentation and cortical hyperexcitability indicates how vision loss disrupts neural input to the visual cortex, leading to compensatory hyperactivity in these brain regions. This excessive firing generates spontaneous visual perceptions, resulting in complex hallucinations despite the absence of real stimuli. Based on the last theory, sensory deprivation theory, reducing visual input provokes a drastic neuron discharge in the occipital lobe could cause visual hallucinations which perfectly aligns with our scenario as in our patient the reduced visual acuity happened due to optic neurites or her occipital lesion in which may affect vision [12].

This disorder is linked to a variety of risk factors, including social isolation and a high level of education, which in our case, our patient was a dentist and due to her physical deterioration became socially isolated [2].

Despite lack of extensive data on CBS epidemiology, experts estimate that more than 10% of patients with visual loss may suffer from this condition. This matter could be explained by two causes: firstly, there is a severe lack of lack of awareness which causes low index of suspiciousness in clinicians. Secondly, patients who are not suffering from psychological problems, due to social stigma of psychiatric disorders, may not visit physicians for a definite diagnosis [2]. In our case our patient Despite her own medical background (dentist), had delay in reporting symptoms which caused her sever stress and agitation. This supports assertions that CBS may disproportionately affect well‐educated individuals, who may deny their symptoms despite their medical knowledge.

Due to the severe lack of knowledge on this syndrome, there are extreme limitations to treatment strategies in this condition and there has yet to be any specific treatment for CBS. Most therapeutic strategies tend to concentrate on vision care and psychological support. Improving vision could increase sensory sensation and thus reduce the symptoms of this syndrome. Even though electromagnetic therapy showed temporary symptom, it has yet to become official treatment for this syndrome [6, 10]. In our case olanzapin were added to patients medication regimen for visual hallucinations. Carbamazepine had been priorly used for CBS treatment by Kurt Segers [13]. A literature review on current treatments on MS and CBS are listed in Table 1.

**TABLE 1:** A summary of current literature on co‐occurrence of MS and CBS.

Age	Sex	Condition	Comorbidities	Management	Outcome	References
56	Female	Hallucinations started 4 months after an episode of optic neuritis	MS	Carbamazepine	visual hallucinations were significantly reduced	[14]
66	Female	Hallucinations started after going completely blind due to optic neuritis after a first attack of MS	MS‐ Diabetes mellitus	—	Hallucination disappeared after vision recovery	[9]
40	Female	Two week hallucinations after 20 years of MS	MS	Olanzapine	Resolvation of hallucinations after 4 weeks	[6]

To sum up, the co‐occurrence of SPMS and CBS presents a complex and extremely unexplored subject within neuro‐psychology field. Due to the probable visual impairment in SPMS patients it is necessary for physicians to perform a comprehensive clinical examination and consider such rare syndromes in patients. Further studies can improve our understanding of the syndromes etiology and connected comorbidities, with the goal of optimizing treatment and patient care.

## Author Contributions

**Shima Jahani:** conceptualization, data curation, formal analysis, investigation, project administration, resources, writing – original draft, writing – review and editing. **Atefeh Eidi Ardizi:** conceptualization, investigation, supervision, writing – review and editing. **MohammadAli Sahraian:** investigation, methodology, project administration, resources, supervision, writing – original draft, writing – review and editing.

## Ethics Statement

The current study did not require ethical approval in accordance with local ethical guidelines. The study was conducted in accordance with the Helsinki Declaration, and informed consent was obtained from patients to discuss or publish the details of their disease, their images, and their course of treatment.

## Consent

Written informed consent was obtained from the patient for publication of this case report.

## Conflicts of Interest

MohammadAli Sahraian: I have received educational, research grants, lecture honorarium, travel supports to attend scientific meetings from Biogen‐Idec, Merck‐Serono, Cinnagen, Zistdaru, Zahravi and Genzyme. Others declare no conflicts of interest.

## Data Availability

The data used to support the findings of this study are included within the article.
